# Poor Oral Hygiene and High Levels of Inflammatory Cytokines in Saliva Predict the Risk of Overweight and Obesity

**DOI:** 10.3390/ijerph17176310

**Published:** 2020-08-30

**Authors:** Kacper Nijakowski, Anna Lehmann, Rafał Rutkowski, Katarzyna Korybalska, Janusz Witowski, Anna Surdacka

**Affiliations:** 1 Department of Conservative Dentistry and Endodontics, Poznan University of Medical Sciences, 61-701 Poznań, Poland; annalehmann@ump.edu.pl (A.L.); annasurd@ump.edu.pl (A.S.); 2 Department of Pathophysiology, Poznan University of Medical Sciences, 61-701 Poznań, Poland; rrutkowski@ump.edu.pl (R.R.); koryb@ump.edu.pl (K.K.); jwitow@ump.edu.pl (J.W.)

**Keywords:** obesity, oral health, oral hygiene

## Abstract

The study aimed to determine if oral hygiene influences not only oral health but also potentially metabolic disorders such as overweight or obesity. Participants were 94 patients: 40 with increased body mass and 54 with normal body mass. The methods included dental examination, a questionnaire concerning hygienic habits and an assessment of selected salivary inflammatory markers. The new parameter named “cleaning index” (describing the interaction between average time of tooth brushing in minutes and its frequency per day) significantly correlated with Body Mass Index (R_Spearman_ = 0.300). The multivariate regression model incorporating cleaning index, approximal plaque index, receptor 1 for tumor necrosis factor-alpha (TNFα-R1) and interleukin-15 (IL-15) had a high power to predict overweight or obesity (AUC = 0.894). Patients with poor oral hygiene (approximal plaque index >40%) were more than eight times more likely to suffer from obesity than patients with good oral hygiene. Cleaning index higher than 4 decreased the odds by about 85%. Oral hygiene habits, adjusted by salivary concentrations of selected inflammatory markers may allow predicting effectively overweight or obesity risk. Early proper dental prophylaxis and treatment could lead to the better prevention of metabolic disorders.

## 1. Introduction

Obesity is a chronic disease with a strong tendency to family history. It affects not only adults but also children and adolescents. In recent years, according to the World Health Organization, obesity has reached pandemic proportions [1]. The most common causes of the disorder are an excessive intake of food, mainly highly processed carbohydrates, and lack of physical activity.

Substances secreted by adipose tissue are present not only in blood but also in saliva [2,3,4,5,6]. Many scientific reports indicate that in addition to the systemic effect, obesity also has a negative impact on the oral cavity. The relationship between overweight and tooth decay and periodontitis is being discussed among researchers [7,8,9]. More and more scientific reports point to changes in the microflora of the oral cavity in the course of obesity. The research by Goodson et al. [10] seems very interesting, who are trying to answer the question of whether oral cavity bacteria are capable of causing obesity. Bacterial DNA was isolated and identified from saliva samples. It turned out that in the saliva of 98.4% of obese women, the presence of *Selenomonas noxia* was detected. The authors put forward a hypothesis about the induction of inflammatory processes leading to obesity with these bacteria.

Poor dietary habits or ubiquitous carbohydrates added to most food products are, in a way, the common denominator of 21st-century diseases. Both obesity and oral diseases are multifactorial and multidimensional. Many overlapping factors are needed for their development. It is obvious that obese patients have an increased and more frequent supply of food, often of little nutritional value and rich in simple sugars and fats. Many researchers also describe the deterioration of oral hygiene and reduced saliva flow in this group. All of these parameters are factors contributing to a higher incidence of dental caries [11,12,13].

Our study aimed to assess if oral hygiene care influences not only on oral health status, but also potentially on metabolic disorders such as overweight or obesity.

## 2. Materials and Methods 

The study group comprised 94 patients (aged 20–54 years, including 57 women) from Department of Internal Diseases, Metabolic Diseases and Dietetics (*n* = 40, with increased body mass) and Department of Conservative Dentistry and Periodontology (*n* = 54, with normal body mass) at the Poznan University of Medical Sciences. None of the patients suffered from coexisting systemic diseases. All patients were measured and weighted to determine values of Body Mass Index (BMI). Table 1 presents the distribution of BMI values for this group according to intervals established by the World Health Organization (WHO).

The patients were asked to answer a questionnaire regarding oral hygiene habits, especially the average time of single tooth brushing (in minutes) and its frequency per day. Based on these two parameters, cleaning index was calculated as their interaction—it was equal to the product of them and thus represented the total number of minutes spent on brushing during the day. Moreover, a clinical examination was performed to assess selected parameters of dental (decayed, missing or filled teeth, DMF-T; decayed, missing or filled surfaces, DMF-S—according to WHO criteria), oral hygiene (approximal plaque index, API; plaque index, PlI) and periodontal (gingival index, GI; sulcus bleeding index, SBI; periodontal probing depth, PPD) status, according to routine methods using mirror, dental probe and periodontal probe WHO-621 (Hu-Friedy Mfg. Co., US) in artificial light. All patients were examined by the same investigator (A.L.). Included patients were free from active caries and periodontitis.

After examination, unstimulated whole mixed saliva was collected for 20 min using the Falcon tubes (volume 50 mL), at the same time of day between 11 a.m. and 1 p.m. Patients were instructed to refrain from eating and drinking at least 2 h before the collection, and to avoid swallowing during it. Subjects were seated comfortably with their eyes open, head tilted slightly forward and their saliva was dripping from the lower lip to the tube located in the container with ice. Then, saliva samples were centrifuged for 10 min with 2000 rpm using Centrifuge MPW-223e (MPW Med. Instruments, Poland), aliquoted into tubes, and stored at −80 °C until assayed. Salivary concentrations of 7 selected markers associated with inflammation (receptor 1 and receptor 2 for tumor necrosis factor-alpha, TNFα-R1 and TNFα-R2; pentraxin 3, PTX-3; interleukin 15, IL-15; monocyte chemoattractant protein-1, MCP-1; soluble intercellular adhesion molecule-1, sICAM-1; soluble CD40 Ligand, sCD40L) were measured with high-sensitivity ELISA immunoassays (DuoSet Immunoassay Development Kit—R&D Systems, Minneapolis, Minnesota, USA) according to instructions.

A statistical analysis was performed using software STATISTICA 13.3 (StatSoft, Cracow, Poland). Due to the lack of normal distribution (assessed by the Shapiro–Wilk test *p* < 0.05), the data were presented as medians and analyzed with nonparametric statistics using the Mann–Whitney test to compare two unpaired groups. The association between selected parameters was evaluated with the Spearman rank correlation and logistic regression. The significance level was defined as α = 0.05 for all analyses.

The study was approved by the Poznan University of Medical Sciences Bioethics Committee (No. 189/14). All patients gave their written informed consent. Every procedure performed in studies involving human participants was in accordance with the 1964 Helsinki declaration and its later amendments, or comparable ethical standards.

## 3. Results

### 3.1. Basic Statistics

The new parameter, named “cleaning index” (describing the interaction between average time of tooth brushing in minutes and its frequency per day) showed significant correlations with other selected parameters evaluated in the study. The index values were observed to decrease with increasing age (R_Spearman_ = −0.418, *p*-value < 0.001) and increasing BMI (R_Spearman_ = −0.300, *p*-value = 0.003), and less significantly with increasing WHR (R_Spearman_ = −0.191, *p*-value = 0.065). Moreover, the higher the cleaning index was, the lower the values were achieved by dental parameters, such as DMF-T, DMF-S and PPD. In turn, a significant increase in API values was observed with increasing BMI (R_Spearman_ = 0.324, *p*-value = 0.002) and increasing WHR (R_Spearman_ = 0.232, *p*-value = 0.028), and in plaque index values only with increasing BMI (R_Spearman_ = 0.342, *p*-value < 0.001). The parameters plaque index and API correlated strongly positively with each other as well as with the gingival index. 

Table 2 and Table 3 present the medians of basic clinical and biochemical parameters determined in the study groups and results of comparison as *p*-values for the Mann–Whitney test.

### 3.2. Logistic Regression Models

**Figure 1:**
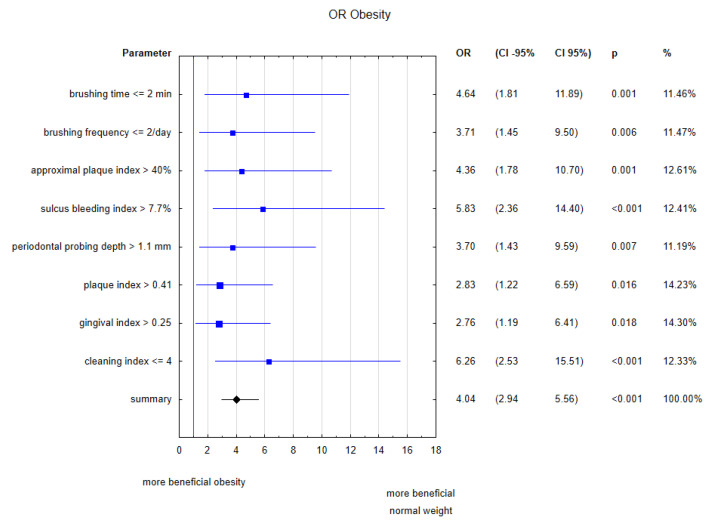
Forest plot of assessed dental parameters in patients with poor oral hygiene concerning odds ratios for obesity.

In order to confirm the influence of oral health on metabolic disorders such as obesity, the univariate logistic regression for assessed dental parameters was demonstrated on forest plot with odd ratios (Figure 1). Binominal categories for these indices were created based on the technique weight of evidence (in relation to the dependent variable—categorical BMI). The most potent risk factor for being in the obese group was the cleaning index equal to or less than 4, directly related to oral hygiene habits.

To better explain the association between oral health and obesity, two logistic regression models were constructed, which take into account predictors with the highest predictive values of d-Somers coefficient, significantly improving their quality. Both models include the approximal plaque index (as a categorical variable), TNF-R1 and IL-15 concentrations. Still, they differ in parameters of oral hygiene habits regarding cleaning index or brushing frequency per day (as categorical variables). Table 4 and Table 5 show the parameters of the predictors incorporated in the models. The calculated odds ratios indicate that the higher API values increase the chance of obesity prevalence to the control group with normal weight—even about eight times. Moreover, higher cleaning index reduces the odds by 85% and only brushing frequency per day by 58%.

**Figure 2:**
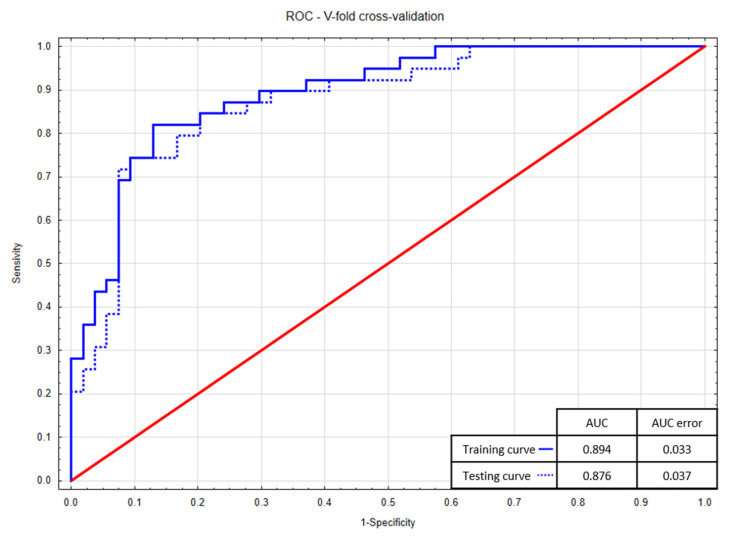
V-fold cross-validation—learning ROC curves for the logistic regression model including cleaning index.

**Figure 3:**
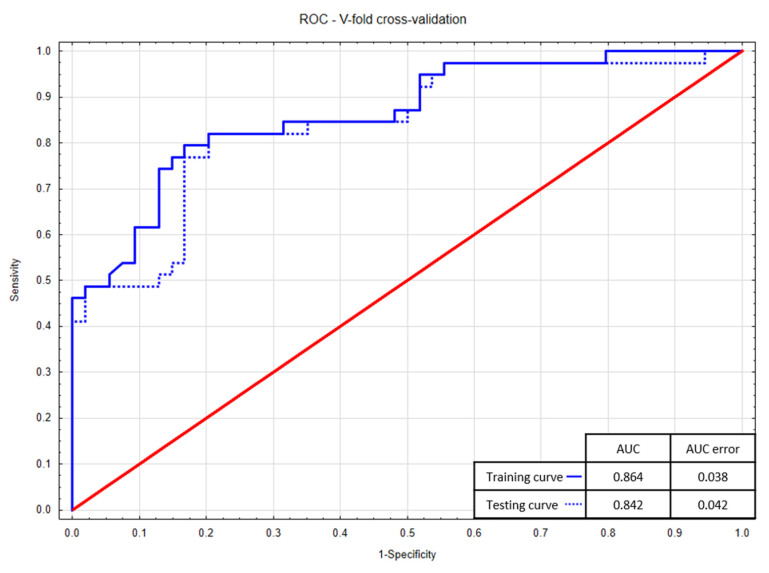
V-fold cross-validation—learning ROC curves for the logistic regression model including brushing frequency per day.

The Hosmer–Lemeshow test was used to assess the goodness of fit—*p*-values higher than 0.05 indicate that the models are well fitted (0.77 vs. 0.25, respectively). The models also have a relatively low Akaike Information Criterion—the regression model incorporating the cleaning index has lower AIC (84.6 vs. 93.9). In addition, the models were validated by the v-fold cross method, and the ROC curves (Figure 2 and Figure 3) for the training (blue solid lines) and testing (blue dotted lines) sample were obtained. The high values of the area under the curve for the training curves (AUC = 0.894 vs. 0.864) and the small differences in areas between the training and testing curves (<0.025) confirm the excellent quality of the models—again, the logistic regression model including cleaning index is better.

### 3.3. Comparative Analysis

**Figure 4:**
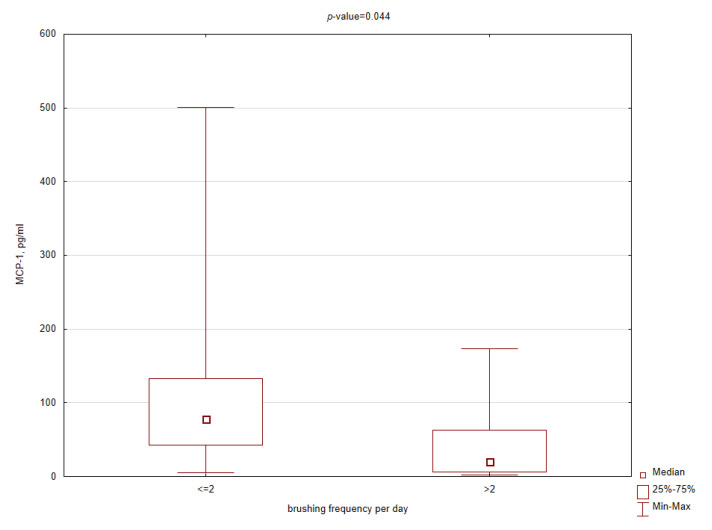
Box plot for salivary monocyte chemoattractant protein 1 (MCP-1) concentrations in the obese patients related to brushing frequency per day (*p*-value < 0.05 according to the Mann–Whitney test).

**Figure 5:**
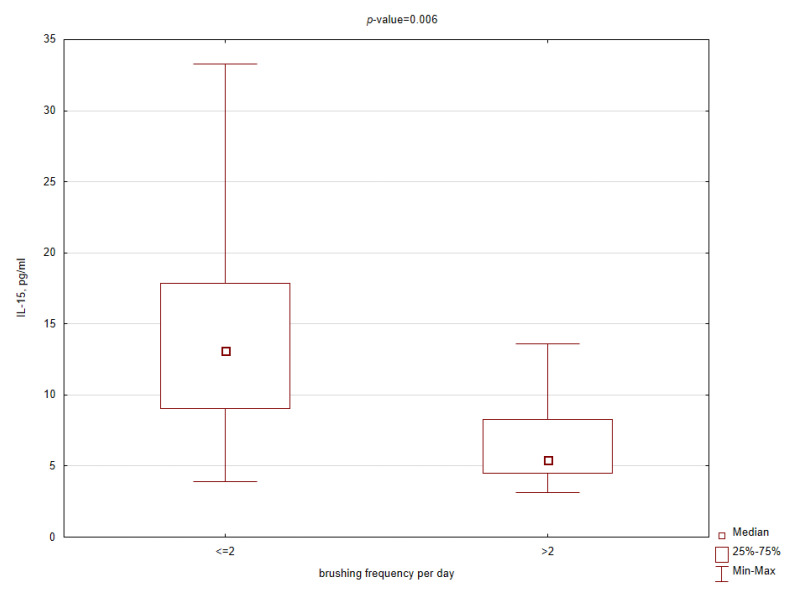
Box plot for salivary interleukin 15 (IL-15) concentrations in the obese patients related to brushing frequency per day (*p*-value < 0.05 according to the Mann–Whitney test).

In addition, among selected salivary inflammatory markers, a significant decrease in concentration in the obese patients brushing more than twice a day was observed only for MCP-1 and IL-15 (Figure 4 and Figure 5). Similar relationships were not found related to higher cleaning index and longer brushing time, suggesting that frequency is more effective than time. In the control group, there were no significant differences in cytokine concentrations depending on oral hygiene habits.

## 4. Discussion

The secretory activity of the adipose tissue and its effect on individual cells, tissues, and organs have been of interest to researchers lately. An excess of adipose tissue, particularly of the visceral type, has been demonstrated to promote and modify the course of periodontitis [8,9]. It results in a higher incidence of dental caries and susceptibility to periodontal diseases. Moreover, the reduced amount of secreted saliva in individuals with obesity was shown to cause changes in the microflora of the oral cavity [14].

Apart from the deteriorated oral health of patients with obesity, a lower volume of secreted saliva was also observed in this group [15]. Saliva plays essential roles in cleaning the oral cavity by removing food remnants and bacteria. Higher salivary flow rate increases oral pH, promotes enamel remineralisation, buffer capacity, and reduces caries. Therefore, a decrease of the saliva flow significantly restricts its protective properties. Previously published studies have shown that, in individuals with obesity, there is moderate persistent underlying inflammation in the parotid glands. It was coupled with inflammatory mediators secreted by adipose tissue and acting along the HPA axis, which could be contributing to the reduced activity of salivary glands [16]. Modéer et al. [17] proposed a hypothesis on the relationship between obesity, reduced salivary flow, and increased incidence of dental caries in young individuals, which later manifests as one of the negative sequels of obesity in the oral cavity in adulthood. As already mentioned above, the development of dental caries in individuals with obesity is considerably affected by the composition and amount of secreted saliva. Its reduced secretion corresponds to the increased accumulation of dental plaque and deterioration of oral cavity hygiene. This study has shown that, in people with high BMIs (>25 kg/m^2^), more severe dental caries was noted. Additionally, sociodemographic factors such as patient’s age may also affect oral health and saliva secretion. As a result of the aging processes and decreased saliva flow rate, the oral cavity becomes more susceptible to caries, periodontal diseases and mucosal problems. However, in our study, age was not determined as a confounder significantly influencing on the amount of saliva or the condition of the oral cavity.

Nevertheless, not only tooth decay is a negative consequence of obesity. Chronic gingivitis or periodontitis is one of the most prevalent diseases of an inflammatory-destructive background and, similarly to obesity, it is classified as a social disease [18]. We found benign gingivitis in both the study and control groups, as shown by the GI and SBI. A previous study by Range et al. [19] showed moderate inflammatory conditions in the gingivae of people with obesity and benign gingivitis in patients with normal body weight. They reported a GI index of 1.95 in the study group and 0.51 in the control group. The critical variable which promotes the development of gingivitis involves the presence of bacteria and their interaction with multiple markers of the host-induced inflammatory process. Cells of adipose tissue produce several cytokines and hormones which increase the risk or alter the course of periodontitis [20]. The measurement of the PPD is also used in evaluating periodontal conditions. The median PPD values were 1.1 mm and 0.8 mm in the study and control group, respectively. We found a statistically significant difference between these values. 

Our study demonstrated and confirmed an apparent relationship between augmented body weight and poor oral hygiene, or the manifestation of gingivitis, or an increased number of teeth affected by dental caries. It has been observed that patients with obesity increase their amount and frequency of food consumed with no appropriate breaks, leading to the continuous accumulation of biofilms, thereby necessitating proper and frequent dental hygienic procedures. Nevertheless, numerous studies indicate that most individuals with obesity have insufficient oral care and inappropriate dental follow-ups. Both Hujoel et al. [21] and Konopka et al. [22] pointed out the lack of flossing of interdental spaces in patients with obesity. While the presence of dental deposits did not in any way correlate with the intensity of caries and the number of teeth, it generated the occurrence of extensive gingivitis. Furthermore, obesity is typically related to unhealthy lifestyle behaviors, and oral health habits (particularly frequency of tooth brushing) is considering as an indicator of general health-related behaviors. In addition to many somatic diseases that affect a significant deterioration in the quality of life of obese patients, there is also a psychological problem. Obese people often suffer from depression caused by social rejection and stigma. These mentioned factors, together with decreased physical fitness and dexterity, may be reasons why they pay less attention to regular oral hygiene. The importance of proper hygienic and dietary habits should be emphasized during developing health promotion programs, aimed at preventing obesity already among children and young people [23].

Overweight and obese patients have a greater chance of being affected by gingivitis due to a combination of metabolic and inflammatory profiles and a neglected attitude towards oral hygiene [24,25]. In earlier studies, we speculated that changes in the salivary cytokine concentrations may have clinical implications for distinguishing obesity-linked comorbidities on oral health [26]. Porcelli et al. [27] reported a positive impact of the oral health preventive program in patients who underwent gastroplasties, contributing to their quality of life. Our study demonstrated statistically significant differences between the indices describing hygienic conditions of the oral cavity in individuals with overweight or obesity and those with normal body weights. The analysis of PlI and API indices showed satisfactory hygiene in both groups. Nevertheless, the median API indicated that the study group had an overall more inadequate oral hygiene when compared with the control group (80% vs. 40%) and the median PlI was 0.7 and 0.3, respectively. Poor oral hygiene may be strongly associated with higher odds of predisposition to overweight or obesity, as suggested by our regression models incorporating the cleaning index and approximal plaque index. Constructed models allow predicting overweight or obesity risk connected with inappropriate oral hygiene habits.

## 5. Conclusions

Our results demonstrated that it is possible to confirm the hypothesis that adipose tissue metabolites interact with the clinical conditions of the oral cavity, strongly related to individual hygienic habits. Perhaps it will allow for the implementation of systemic solutions that would enable appropriate steps to be taken to increase dental care for overweight and obese patients, both in terms of prevention and treatment.

## Figures and Tables

**Table 1:** Distribution of Body Mass Index in patients.

Category	BMI, kg/m^2^	*n*
Underweight	<18.5	6
Normal	[18.5–25)	48
Overweight	[25–30)	11
Obese class I	[30–35)	13
Obese class II	[35–40)	9
Obese class III	>40	7

**Table 2:** Medians of assessed dental parameters in patients.

Parameter	All	BMI < 25	BMI ≥ 25	*p*-Value
DMF-T	9.5	8.5	11.0	0.002 *
DMF-S	13.0	11.0	19.5	<0.001 *
Approximal plaque index, %	60.0	40.0	80.0	<0.001 *
Plaque index	0.4	0.3	0.7	0.003 *
Gingival index	0.3	0.1	0.5	0.013 *
Sulcus bleeding index, %	7.7	1.9	15.4	<0.001 *
Periodontal probing depth, mm	0.9	0.8	1.1	0.029 *
Brushing frequency/day	2.0	2.0	2.0	0.004 *
Brushing time, min	2.0	3.0	2.0	0.010 *
Cleaning index	6.0	6.0	4.0	<0.001 *

* Significant difference for *p*-value < 0.05 according to the Mann–Whitney test.

**Table 3:** Medians of assessed salivary concentrations of inflammatory cytokines.

Cytokine, pg/mL	All	BMI < 25	BMI ≥ 25	*p*-Value
TNFα-R1	154.6	98.4	230.6	<0.001 *
TNFα-R2	53.0	42.6	71.1	0.026 *
PTX-3	314.2	246.5	386.8	0.005 *
IL-15	9.9	9.0	12.0	0.012 *
MCP-1	39.3	20.0	62.6	0.007 *
sICAM-1	418.1	325.8	596.9	0.002 *
sCD40L	4.6	4.7	3.1	0.003 *

* Significant difference for *p*-value < 0.05 according to the Mann–Whitney test.

**Table 4:** Parameters of predictors incorporated into the multivariate logistic regression model, including cleaning index.

	β	SE	WaldStat.	*p*-Value	Odds Ratio	Confidence OR −95%	Confidence OR 95%
intercept	−3.537	1.054	11.269	**<0.001**	0.029	0.004	0.229
cleaning index > 4	−1.913	0.601	10.128	**0.001**	0.148	0.045	0.480
API > 40%	2.087	0.678	9.471	**0.002**	8.061	2.134	30.457
TNF-R1, pg/mL	0.008	0.002	11.402	**<0.001**	1.008	1.003	1.012
IL-15, pg/mL	0.136	0.054	6.350	**0.012**	1.145	1.031	1.273

Bold variables with significant difference for *p*-value < 0.05.

**Table 5:** Parameters of predictors incorporated into the multivariate logistic regression model, including brushing frequency per day.

	β	SE	Wald Stat.	*p*-Value	Odds Ratio	Confidence OR −95%	Confidence OR 95%
intercept	−4.165	1.048	15.788	**<0.001**	0.016	0.002	0.121
cleaning index > 4	−0.858	0.609	1.983	0.159	0.424	0.128	1.400
API > 40%	2.012	0.627	10.296	**0.001**	7.476	2.188	25.550
TNF-R1, pg/ml	0.007	0.002	11.350	**<0.001**	1.007	1.003	1.011
IL-15, pg/ml	0.145	0.051	8.244	**0.004**	1.157	1.047	1.277

Bold variables with significant difference for *p*-value < 0.05.
